# Editorial: Organization and Functional Properties of the Blood-Brain Barrier

**DOI:** 10.3389/fphys.2021.796030

**Published:** 2021-12-02

**Authors:** Darryl R. Peterson, Richard A. Hawkins, Juan R. Viña

**Affiliations:** ^1^Discipline of Physiology and Biophysics, Chicago Medical School/Rosalind Franklin University of Medicine and Science, North Chicago, IL, United States; ^2^Department of Biochemistry and Molecular Biology, Faculty of Medicine, Institute INCLIVA, University of Valencia, Valencia, Spain

**Keywords:** structure, transport, drug delivery, disease, blood-brain barrier

## Introduction

This Editorial provides a brief overview of the organization and functional properties of the blood-brain barrier, and introduces the foundational information in this book that contributes to these principles.

## The Organizational Role of the Blood-Brain Barrier

The blood-brain barrier serves to restrict and control passage of solutes between the general circulation and brain extracellular fluid (Abbott and Friedman, 2012). Barrier function is due principally to the presence of specialized endothelial cells that line brain capillaries (Ge et al., 2005). These endothelial cells possess tight junctions that circumscribe the cells and largely prohibit extracellular movement of solutes between the cells (Hawkins and Davis, 2005; Tornavaca et al., 2015; Sweeney et al., 2019). The tight junctions demarcate two distinct plasmalemmal domains within the endothelium, including the luminal (blood facing) and abluminal (brain facing) plasma membranes (Sanchez del Pino et al., 1995; Peterson and Hawkins, 2003). Thus, transport across the blood-brain barrier is primarily transcellular, and net movement of solutes across the endothelium is determined by transport properties of the respective plasma membrane domains (Peterson and Hawkins, 1998, 2003; Hawkins et al., 2002). These substances may be altered by degradative enzymes associated with the barrier (el-Bacha and Minn, 1999), and barrier function is influenced by adjacent cells including astrocytes and pericytes that have led to the concept of a “neurovascular unit” (Hawkins and Davis, 2005; Abbott et al., 2006; Armulik et al., 2010; Sweeney et al., 2019).

## Functions of the Blood-Brain Barrier

The blood-brain barrier contributes to homeostatic control of the central nervous system by modifying the volume and composition of brain extracellular fluid (Strange, 1992; Keep et al., 1998; Abbott and Friedman, 2012; Sweeney et al., 2019). Specific carrier proteins are present in both the luminal and abluminal plasma membranes of brain capillary endothelial cells that allow facilitated passive transport of nutrients from blood to brain, including glucose and amino acids (Drewes, 1998; Smith and Stoll, 1998; Hawkins et al., 2011; Peterson, 2019). Some amino acid transport systems possess sodium-dependent (i.e., secondary active) carriers in the abluminal membrane, thus providing possible mechanisms for regulation of transport (O'Kane et al., 1999; Peterson, 2019). Although the blood-brain barrier appears to be largely impermeable to peptides and proteins, there is evidence that some small peptides may enter the brain utilizing carrier-mediated processes (Banks, 2015; Sweeney et al., 2019). Furthermore, it appears that insulin and transferrin may cross the barrier by receptor-mediated endocytosis (Duffy and Pardridge, 1987; Pardridge et al., 1987). Unidirectional (blood-to-brain) fluid movement across the blood-brain barrier utilizes coordinated transport of salt and water that appears to be regulated (Strange, 1992; Keep et al., 1998; Peterson and Hawkins, 1998; Abbott and Friedman, 2012; Peterson, 2019; Sweeney et al., 2019). Sodium first enters passively into the cells utilizing carriers (e.g., Na/H antiporter, Na/K/Cl cotransporter, Na/Ca exchanger) in the luminal membrane of brain capillary endothelial cells. Intracellular sodium is then actively pumped out across the abluminal membrane by a Na/K-ATPase. It is generally believed that water passively follows sodium transport by utilizing water channels (i.e., aquaporin) in both the luminal and abluminal membranes (Nagelhus and Ottersen, 2013). There is evidence that fluid transport is regulated by centrally released peptide hormones (Strange, 1992; Abbott et al., 2006), and that normal fluid balance is achieved by its uptake and drainage into and out of the central nervous system.

## Drug Delivery Across the Blood-Brain Barrier

One of the fundamental challenges to developing drugs that target the brain is the presence of the blood-brain barrier (Pardridge, 2012). However, understanding the mechanisms by which solutes are transported by blood-brain barrier endothelial cells provides a basis for designing drugs that are capable of traversing the barrier (Sweeney et al., 2019). For instance, pharmacological agents that are recognized by transport carriers that normally deliver nutrients to the brain would likely cross the blood-brain barrier. Another potential pathway involves utilization of normally occurring transcytotic pathways (Pardridge, 2012). Thus, coupling pharmacological agents that bind insulin or transferrin receptors has been shown to mediate drug delivery across the blood-brain barrier (Pardridge, 2012). In addition, procedures designed to loosen tight junctions have been used to promote intercellular movement of drugs across the barrier (Rapaport, 2000; Hsu et al., 2018). Nevertheless, each of these procedures for enabling passage of drugs across the blood-brain barrier is complicated by the presence of active efflux transporters in the endothelial cells that may limit or prevent net influx (Schinkel, 1999; Abbott et al., 2006; Reichel et al., 2011; Sweeney et al., 2019).

## Alterations of the Blood-Brain Barrier in Disease

There is now evidence that dysfunction of the blood-brain barrier accompanies several diseases involving the central nervous system (Sweeney et al., 2019). For instance, blood-brain barrier dysfunction has been associated with Alzheimer's disease, amyotrophic lateral sclerosis, epilepsy, multiple sclerosis, Parkinson's disease, stroke, and traumatic brain injury (Papadopoulos et al., 2001; Lo et al., 2003; Marroni et al., 2003; Minagar and Alexander, 2003; Lee and Bendayan, 2004; Kortekaas et al., 2005; Peterson and Sukowski, 2019). Alterations of the barrier include changes in its permeability, transport properties, and regulatory mechanisms. Thus, understanding the normal structure and function of the blood-brain barrier, and defining the properties that are altered during pertinent neurological disorders, could provide a logical approach to designing effective therapeutics.

## Book Chapters

The chapters in this book serve to: (1) review important discoveries in defining the organization and functional properties of the blood-brain barrier, and (2) introduce new concepts regarding its normal function or participation in disease processes.

The presentation by Partridge describes significant contributions of using brain microvessels as a model for investigating the blood-brain barrier and the neurovascular unit. Isolated brain microvessels consist of endothelial cells, pericytes, pre-capillary arteriolar smooth muscle cells, astrocyte foot processes, and nerve endings. They have been used as an *in vitro* model of the blood-brain barrier to: (1) produce cDNA libraries for genomic analyses, (2) quantify the presence of specific transporters and receptors using proteomics, (3) determine the cellular location of proteins expressed within the neurovascular unit by using immunolabeling, (4) study kinetic parameters of transport carriers, and (5) quantify dissociation constants of peptide binding involved in receptor-mediated transport.

The article by Locchead et al. focuses on functional properties of tight junctions in the blood-brain barrier, and how these are altered during certain pathological conditions. This presentation also describes how the blood-brain barrier may be manipulated therapeutically to allow for intercellular delivery of systemically administered drugs to the brain. Molecular elements of tight junctional function, regulation, and potential therapeutic manipulation are defined.

The paper by  Brunner et al. describes a technique to quantify the contribution of claudins in the seal characterized by blood-brain barrier tight junctions. *Xenopus lauvis* oocytes are used as an expression system for claudins, and homophilic and heterophilic trans-interactions are characterized. The effect of hydrostatic pressure on the stability of cell-to-cell connections and their modulation are quantified as a function of claudin expression. This technique provides a potential basis for a more complete understanding of tight junction function and control in the blood-brain barrier.

The review by Zaragozå gives an inclusive over-view of amino acid transport by the blood-brain barrier. Both facilitative and secondary active transport processes are described, and the functional significance of a polarized distribution of amino acid transporters is discussed.

## Author Contributions

All authors listed have made a substantial, direct, and intellectual contribution to the work and approved it for publication.

## Conflict of Interest

DP (inventor) and Rosalind Franklin University of Medicine and Science (assignee) have been awarded patents that are related to research done on treatment of stroke. They both may benefit financially upon commercialization of the patents. DP is a Professor Emeritus at Rosalind Franklin University of Medicine and Science and owns Harbor Biotechnology LLC, a company that seeks to commercialize the patents referenced above. Further details may be obtained from DP upon request. The remaining authors declare that the research was conducted in the absence of any commercial or financial relationships that could be construed as a potential conflict of interest.

## Publisher's Note

All claims expressed in this article are solely those of the authors and do not necessarily represent those of their affiliated organizations, or those of the publisher, the editors and the reviewers. Any product that may be evaluated in this article, or claim that may be made by its manufacturer, is not guaranteed or endorsed by the publisher.
